# The Importance of Structural Anisotropy in Computational Models of Traumatic Brain Injury

**DOI:** 10.3389/fneur.2015.00028

**Published:** 2015-02-19

**Authors:** Rika W. Carlsen, Nitin P. Daphalapurkar

**Affiliations:** ^1^Department of Engineering, Robert Morris University, Pittsburgh, PA, USA; ^2^Department of Mechanical Engineering, Hopkins Extreme Materials Institute, Johns Hopkins University, Baltimore, MD, USA

**Keywords:** traumatic brain injury, diffuse axonal injury, computational model, injury criterion, axonal strain

## Abstract

Understanding the mechanisms of injury might prove useful in assisting the development of methods for the management and mitigation of traumatic brain injury (TBI). Computational head models can provide valuable insight into the multi-length-scale complexity associated with the primary nature of diffuse axonal injury. It involves understanding how the trauma to the head (at the centimeter length scale) translates to the white-matter tissue (at the millimeter length scale), and even further down to the axonal-length scale, where physical injury to axons (e.g., axon separation) may occur. However, to accurately represent the development of TBI, the biofidelity of these computational models is of utmost importance. There has been a focused effort to improve the biofidelity of computational models by including more sophisticated material definitions and implementing physiologically relevant measures of injury. This paper summarizes recent computational studies that have incorporated structural anisotropy in both the material definition of the white matter and the injury criterion as a means to improve the predictive capabilities of computational models for TBI. We discuss the role of structural anisotropy on both the mechanical response of the brain tissue and on the development of injury. We also outline future directions in the computational modeling of TBI.

## 1 Introduction

Computational models incorporating high-fidelity tissue-level anatomy and the mechanical response of various tissues have become a valuable tool for studying the development of traumatic brain injury (TBI). One of the most common pathological features in mild traumatic brain injury is diffuse axonal injury. It has been hypothesized that this injury occurs as a result of the stretching of axons (1). The white matter of the brain contains axons, which are bundled into fiber tracts and serve as communication pathways in the brain. The coherent orientation of fibers in white matter, which lead to the anisotropy in the mechanical response of the white matter, plays an important role in the development of injury. Understanding, through experiments, the relationship between tissue-level loading and axonal injury is imperative to develop constitutive models that incorporate the mechanical response under various loading conditions, and to further use these models to develop appropriate measures of injury. Computational models thus provide a platform for integrating various mechanical and even biochemical models with the anatomy of the brain. Applications of such a computational platform include the ability to predict local deformations and stresses, the likelihood of primary injuries (predominantly with physics-based models), secondary injuries (with inclusion of biochemical models), the likelihood of neurologic outcomes that might occur in diffuse-axonal-injury-related TBI, and the development of kinematic tolerance thresholds for safeguarding from brain injury.

A specific application of such a computational platform includes predicting the severity of primary injury immediately following a TBI event (2). A functional axonal injury criterion that is based on stretch can be used to predict the likelihood of primary injury to the axons (that is instantaneous), which considers the collective effect of multiple mechanisms, e.g., increase in membrane permeability, disruption of axonal transport, and axonal separation. A primary injury might further lead to a variety of biochemical cascades and other mechanisms of secondary injury that develop over a longer time duration (3). With the increased use of wearable sensors, such as football helmets instrumented with accelerometers, it has become possible to acquire kinematic data in real-time. Measured kinematic parameters can then be applied as inputs into the model, which can be used to predict the location and extent of injury in the brain for a given injury event, serving as an objective measure for the likelihood of injury. Currently, the diagnosis of mild traumatic brain injury relies heavily on neurocognitive assessments since the structural signature of injury is not visible in conventional medical imaging modalities. The damage might occur predominantly at the cellular level, which is beyond the resolution of many commonly used imaging platforms. In such cases, computational models could serve as an invaluable tool for predicting the likelihood of injury. Used in conjunction with neurocognitive assessments, they could potentially aid in providing guidance on the possible location of injury.

Despite such potential for critical applications, there has been limited success in establishing tolerance thresholds for brain injury using computational models. Furthermore, the ability of computational models to accurately predict the location and severity of axonal damage has yet to be validated. This may be in part due to the need to improve the biofidelity of existing computational models. Many factors contribute to the biofidelity of these models, including the use of appropriate boundary conditions and material definitions, the level of anatomical detail, and accuracy of the injury measure. Recently, there has been a push to apply more physiologically relevant injury criteria and to account for the substructure of the white matter (4). It has been hypothesized that the orientation of the fibers in the white matter play an important role in both the injury development and the mechanical response of the brain tissue.

In an effort to assess the importance of the structural anisotropy in computational models of TBI, we provide a summary of selected studies that have accounted for the structural anisotropy of the white matter in computational head models either through the injury criterion for axonal injury or the material definition of the white matter. We discuss the effect of this structural anisotropy on the prediction of injury and summarize what can be learned about the role of the tissue-substructure in the development of diffuse axonal injury from these studies. Since the inclusion of structural anisotropy can increase the computational cost and complexity of a model, it is important to assess its impact on the biofidelity and predictive capabilities of the model.

## 2 Structural Anisotropy in Computational Head Models of TBI

**Figure 1:**
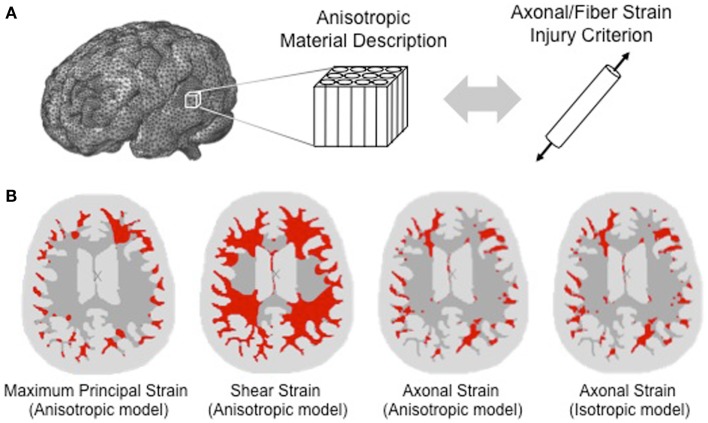
**Accounting for structural anisotropy in a computational head model**. **(A)** Structural anisotropy can be incorporated into computational head models either through an anisotropic material description for the white matter or through an injury criterion that accounts for the fiber orientation, such as stretch along the fiber direction (Brain model image courtesy of Dr. Reuben Kraft). **(B)** Predicted regions of axonal injury for an axial slice of brain are highlighted in red using a finite element analysis of a head impact that resulted in concussive injury. Starting from the left, the first three images show the difference in the predicted location of injury for different injury criteria. Injury thresholds of 31 and 25% were adopted for the maximum principal strain and shear strain, respectively (6), and a strain threshold of 18% was used for the axonal strain (7). With a structurally based injury criterion such as axonal strain, the locations of injury do not differ significantly between anisotropic and isotropic material definitions of the white matter as shown in the third and fourth image of the same axial slice [adapted from Ref. (8) with permission].

The advent of diffusion tensor imaging (DTI) has made it possible to incorporate the structural anisotropy of the white matter into computational models of TBI. DTI measures the diffusion of water molecules in the brain. Since water molecules diffuse faster along fibers than perpendicular to them, the technique can be used to characterize the orientation of axons within a given region of the brain (5). Whereas early computational head models of TBI treated the brain as a homogeneous mass and defined injury based on equivalent stress and strain measures, such as the von Mises stress, many recent computational head models of TBI have accounted for the anisotropy of the brain tissue through the use of diffusion tensor imaging. As shown in Figure 1A and described in the following sections, the structural anisotropy can be accounted for both in the material response of the brain tissue and in the development of axonal injury.

### 2.1 Inclusion of structural anisotropy into the material definition

The white matter is anisotropic in nature due to the coherent orientation of fibers, and this structural anisotropy affects the mechanical behavior of the tissue (9). The material properties of the white matter has been shown to be dependent on the relative direction of loading with respect to the fiber orientation (10, 11). Quasistatic stretch experiments on the brain white-matter tissue by Velardi et al. (12) and data analysis to characterize the effect of fiber reinforcement (7) suggests the shear modulus along the fiber direction is approximately 42% higher compared to the modulus perpendicular to the fiber direction.

In a computational model, data from diffusion tensor imaging (DTI) can be used to assign the local fiber orientation within the white matter. DTI is a magnetic resonance imaging (MRI) technique that provides both a measure of the average orientation of the axons and the degree of fiber dispersion for a local volume element (typically at a resolution of several cubic millimeters). By co-registering a diffusion tensor image of the brain with anatomy data from magnetic resonance imaging, a volume-averaged fiber orientation can be defined for each finite element within a computational model of the brain, which also integrates the data associated with the mechanical response of the white matter. Then, an anisotropic material model can be applied to define the local material response based on this volume-averaged fiber direction for white matter of the brain. The mechanical response of the gray matter can be assumed as predominantly isotropic.

The constitutive model is an important part (of the computational head model) that describes generally the mechanical response of tissues in the brain, taking into account the loading conditions and the tissue-substructure. Unique to such constitutive models is how the strain energy function is defined, what specific substructural features it considers, and assumptions involved that discount any other less important or unsubstantiated phenomena or mechanisms. Several anisotropic hyperelastic strain energy functions have been applied to model the material behavior of the white matter, including the quadratic reinforcing strain energy function (7), the Holzapfel-Gasser-Ogden (HGO) model (8, 13–15), and a strain energy function by Puso and Weiss (16, 17). In particular, the HGO model (that was originally developed for collagenous tissue) has been generalized to account for the fiber dispersion in brain white matter, rather that treating the fibers as perfectly aligned. Wright et al. (8) and Giordano et al. (15) demonstrated the application of a simplified version of the HGO model to brain, by reducing the number of material parameters that can be characterized from available experiments. From Wright et al. (8), assuming a single family of fibers, the strain energy function for the HGO model simplifies to

(1) $$W=\frac{\mu }{2}\left(\bar{I_{1}}-3\right)+\frac{k_{1}}{2}{\left\langle {\bar{E}}_{1}\right\rangle }^{2}+\frac{K}{2}\left(\frac{J^{2}-1}{2}-\ln J\right),$$

where *μ* is the shear modulus, $\bar{I_{1}}=\mathrm{tr}\bar{C}$ is the first invariant of the deviatoric component of the right Cauchy-Green deformation tensor, $\bar{C},$ from continuum mechanics, $\bar{E_{1}}=\kappa \left(\bar{I_{1}}-3\right)+\left(1-3\kappa \right)\left(\bar{I_{4}}-1\right)$ is the term that incorporates the fiber dispersion parameter (*κ*), *J* is the Jacobian of the deformation gradient tensor, *k*_1_ is the degree of fiber reinforcement and *K* is related to the bulk modulus. A specific case of perfectly aligned fibers (transversely isotropic) can be simulated by setting a fiber dispersion parameter to zero (*κ* = 0).

The fiber dispersion parameter in the HGO constitutive model can be correlated to the fiber dispersion measure obtained from DTI to account for the crossing of fibers. In diffusion tensor imaging, the fiber dispersion is represented by a parameter called the fractional anisotropy (FA), which is computed from the eigenvalues of the diffusion tensor (8, 18). Giordano et al. (18) derived the following relationship between the fiber dispersion parameter (*κ*) and the fractional anisotropy:

(2) $$\mathrm{FA}=\frac{\sqrt{{\left(\left(1-2\kappa \right)∕\left(\kappa \right)\;-1\right)}^{2}}}{\sqrt{{\left(\left(1-2\kappa \right)∕\left(\kappa \right)\right)}^{2}+2}}$$

A similar relationship was also used by Wright et al. (8). By combining the fiber orientation data from DTI with an anisotropic material description for the white matter, the anisotropic behavior of the brain tissue can thus be modeled considering the realistic fiber network.

### 2.2 Implementation of a structurally based injury criterion

In an effort to better predict the likelihood of injury using a computational head model, several studies have incorporated the stretch of axons beyond a critical value as a criterion of injury. To compute the stretch of axons, the direction of neural axon alignment must be known; therefore, the structural anisotropy of the white matter must be incorporated into the computational model. Data from DTI can be used to define the volume-averaged orientation of fibers for various locations within the white matter of the brain. Due to lack of access to high-resolution imaging techniques that could characterize the tissue-substructure, computational models typically assume a direct correlation for measures of strain (or stretch) and orientation between axons and fibers within a local volume element (typically at a resolution of several cubic millimeters). The nominal strain component along the direction of axonal alignment can then be computed. A strain threshold for functional damage can be defined based on experimental studies of axonal injury (3). Bain and Meaney determined an optimal strain threshold of 18% for functional and morphological injury to the optic nerve of a guinea pig subjected to stretch. This injury criterion is representative of the substructural, electrophysiological, and possibly biochemical changes that occur in the fibers due to axonal stretch, which eventually lead to degeneration and functional damage.

This structurally based injury criterion has been termed “axonal strain” or “fiber strain” and has been applied in several computational studies of TBI as a measure of diffuse axonal injury (Table 1). Other variants of the structurally based injury criterion have also been developed. Cloots et al. applied an anisotropic equivalent strain measure in a multi-scale computational model (19). This equivalent strain measure relates the maximum axonal strain within a critical volume element at the micro-level with the deviatoric tissue strain components at the macro-level. One of the advantages of applying these structurally based injury criteria is that they are based on a physiologically relevant mechanism of injury. The hope is that these measures of injury will provide a better prediction of injury than previously used measures, such as the von Mises stress, which is not based on a known mechanism of injury.

**Table 1:** **Examples of computational head models of TBI that incorporate structural anisotropy**.

	Simulated TBI event	Finite element model	Structurally based white matter model	Structurally based injury criterion
Colgan et al. (14)	Head rotation	UCDBTM (3-D)	Anisotropic, visco-hyperelastic	–
Chatelin et al. (20)	2 Reconstructed motorcycle accidents	SUFEHM (3-D)	–	Axonal strain
Kraft et al. (21)	Head impact	Kraft et al. (3-D)	Anisotropic, hyperelastic	Axonal strain (time-evolving)
Cloots et al. (19)	Reconstructed sporting accident	Kleiven (3-D)	Anisotropic, visco-hyperelastic	Axonal strain
Wright et al. (8)	Reconstructed concussive hockey impact	Wright-Ramesh (2-D)	Anisotropic, visco-hyperelastic	Axonal strain
Giordano et al. (15)	2 Reconstructed concussive football impact	KTH (3-D)	Anisotropic, visco-hyperelastic	Axonal strain
Ji et al. (2)	11 Concussive football and hockey impacts	DHIM (3-D)	–	Fiber strain
Sahoo et al. (17)	Head impact	SUFEHM (3-D)	Anisotropic, visco-hyperelastic	–

## 3 Effect of Anisotropy on the Predicted Injury Response

The structural anisotropy of the white matter has been shown to have a significant effect on the prediction of likely injury. Several computational studies have compared the mechanical response of the brain tissue for an isotropic and an anisotropic definition of white matter. Sahoo et al. found that the inclusion of anisotropy had a significant influence on the local brain motion that developed in a simulated head impact (17). The inclusion of anisotropy was found to have a significant effect on the magnitude and direction of the developed principal strains (15) and on the magnitude of the developed shear strain in some fiber tracts of the white matter (14).

To study the impact that a structurally based injury criterion has on predicted injury, several computational head model studies have compared the injury predictions for a structurally based injury criterion with that of traditionally used tissue-level injury criteria, such as the first (i.e., maximum) principal strain, von Mises stress, and shear strain. Chatelin et al. simulated head impacts sustained in two well-documented motorcycle accidents (20). In one of the accidents, severe diffuse axonal injury and subdural hematoma resulted, whereas the other accident resulted in no severe injury. The magnitudes of the axonal strain, von Mises strain, and first principal strain were all found to be 100% higher for the head impact that resulted in injury compared to the non-injurious head impact. For both simulated cases, the axonal strains were significantly smaller (approximately 30%) and were closer in magnitude to experimentally determined axonal injury thresholds (3) compared to the magnitudes predicted by the von Mises strain and the first principal strain. Chatelin et al. further brought to light, if the locations susceptible to injury were to be defined based on the regions that experience the highest magnitude of strain, then differences were observed in the resulting predictions of injured regions between the individual strain measures. The first principal and the von Mises strain predicted injury in the brain periphery, and the axonal strain criterion predicted injury in the white-matter tracts, such as the corpus callosum, which is where diffuse axonal injury is commonly found (22). These trends were confirmed by Wright et al. (7).

Wright et al. simulated the head impact of a professional ice hockey player that resulted in concussive injury (8). Comparison of different measures of deformation as criteria for injury are shown in Figure 1B [from Ref. (8)]. Applying commonly used tolerance thresholds for injury, Figure 1B shows that a much greater degree of damage was predicted with the shear strain and the first principal strain injury criteria than with the axonal strain criterion. The fiber tracts with highest axonal strain correlated (8) with predominant regions of damage that were identified in studies of concussive injury. When using a structurally based tissue measure, such as the axonal strain, Wright et al. found that both isotropic and anisotropic material definitions of the white matter produced similar regions of high axonal strain. It was hypothesized that this similarity was due to the fact that the tissue-substructure was captured through the structurally based injury measure (8). However, for other tissue-level measures of injury, such as the von Mises stress, shear strain, and maximum principal strain, significant differences were found between isotropic and anisotropic material models (7, 8). These sensitivity studies illustrate that when applying strain-based criteria for predicting functional injury in axons, consideration of the strain component along the fiber orientation in the local region of the white matter is crucial.

Ji et al. simulated 11 concussive football and hockey impacts using instrumented helmet acceleration data as inputs into their computational head model and considered strains along the fiber direction as a measure for injury (2). They found significant differences in the distribution and extent of predicted damage between the fiber strain and first principal strain criterion for injury. The distribution of regions with high fiber strain was consistent with typical heterogeneous patterns of diffuse axonal injury. In all these studies, the pattern of injury predicted with a structurally based injury criterion that takes the structural anisotropy of the white matter into consideration was highly consistent with that seen in pathological studies of TBI.

Another important conclusion that can be drawn from these studies is that the development of diffuse axonal injury is highly dependent on the direction of loading. Giordano et al. simulated two concussive football impacts and found that the first principal strain as a criterion for injury over-predicted the extent of injury compared to that of the axonal strain injury criterion (15). The extent of over-prediction was found to be dependent on the loading direction with respect to the orientation of the axonal fibers. Kraft et al. applied a time-evolving injury criterion that was based on both the axonal strain and strain rate (21). They found that the orientation of the fibers with respect to the direction of impact affected the extent of predicted injury. The extent and degree of injury have been shown to be significantly different between linear and rotational accelerations of the head (8). Under general conditions of head impact, it has been hypothesized that the rotational component of acceleration is the dominant contributor to axonal damage. These studies highlight the significance of the loading direction, which has an important implication on the development of kinematic tolerance criteria for diffuse axonal injury. Kinematic tolerance criteria should not only include a tolerance threshold for injury based on the magnitude of acceleration but should also take into consideration the direction of impact.

## 4 Future Directions

At the heart of a predictive computational tool is the mechanics associated with the biology. Computational models in combination with imaging techniques, such as diffusion tensor imaging, have the potential to improve our understanding of injury by providing a framework to integrate different physical models for a variety of injury mechanisms. The ability of computations to predict the complex behavior of these coupled systems needs a closer look from an applications viewpoint. It is critical to be able to correlate predicted injury with actual injury and to train the framework to improve based on quantitative metrics of comparison. It was demonstrated that anisotropy is important and has a significant effect on the predicted injury response. However, whether the inclusion of anisotropy is sufficient to improve the predictive capabilities of a model has yet to be validated. Recently, deformation in the human brain is being quantified *in vivo* using tagged MRI (23) and may be a promising way forward to validate model predictions of brain deformation. Computational models that account for multiple families of fibers within each volume element may also improve the predictive capability. A limitation of current DTI methods is the accurate characterization of two or more families of crossing fibers in a single voxel. As DTI methods improve, higher resolution substructure data can be incorporated into computational models. Another future direction involves using a combination of patient-specific computational head models, event-specific kinematics, and DTI/MRI data for cross-validation of the models with actual patient data. Observations of secondary injury, neurodegeneration over time, and experiments on tissues demonstrating rate effects are also important considerations from a modeling viewpoint to accurately represent the progression of injury.

A noble goal in developing these models is to enable health care professionals to have further insight into the likelihood of diffuse axonal injury. From a physicians viewpoint, clinical diagnosis of concussion with currently available tools may not be enough; one might be able to use physics-based computational tools, in conjunction with imaging methods, to relate predicted locations of injury with actual physical and functional changes. As neuroimaging methods continue to improve, this may be a possibility in the future. Quantifying the likelihood and extent of the injury still remains a significant challenge. Approaches that quantify the likelihood of injury with respect to specific fiber tracts using a white-matter atlas (8) or a connectome (21), which maps neural connections in the brain, may help bridge the gap between the data obtained in a clinical setting with the results from physics-based computational models. Computational models that incorporate structural anisotropy of white matter and the axonal-level injury criterion might lead to much more rigorous and comprehensive set of thresholds for the likelihood of diffuse axonal injury due to head trauma.

## 5 Conclusion

There is still a critical need to improve the predictive capabilities of computational models of TBI so that they can provide better insight into the mechanisms of injury. With improvements in medical imaging techniques and availability of real-time measurements of kinematic data during injury events, we have more resources available to enhance the biofidelity of these computational tools. To improve the representation of the mechanical behavior of brain tissue and the predicted injury response, there has been a push to account for the structural anisotropy of the white matter in computational analyses. Recent studies have shown that this structural anisotropy can have a significant effect on the brain deformation, and the use of a structurally based injury criterion can lead to injury predictions that are more consistent with known patterns of axonal injury. Results suggest that the inclusion of structural anisotropy may be a step in the right direction toward improving the biofidelity of computational models of TBI. The effectiveness of this computational approach would benefit from a higher order validation using high-fidelity experimental measurements of deformation in the brain and cross-validation with actual patient data. As we continue to improve the predictive capabilities of these models, they will serve even greater value in understanding the development of TBI.

## Conflict of Interest Statement

The authors declare that the research was conducted in the absence of any commercial or financial relationships that could be construed as a potential conflict of interest.
